# Probing the intrinsic failure mechanism of fluorinated amorphous carbon film based on the first-principles calculations

**DOI:** 10.1038/srep09419

**Published:** 2015-03-24

**Authors:** Ren-hui Zhang, Li-ping Wang, Zhi-bin Lu

**Affiliations:** 1 State Key Laboratory of Solid Lubrication, Lanzhou Institute of Chemical Physics, Chinese Academy of Science, Lanzhou 730000, China; 2 University of Chinese Academy of Sciences, Beijing 100049, China

## Abstract

Fluorinated amorphous carbon films exhibit superlow friction under vacuum, but are prone to catastrophic failure. Thus far, the intrinsic failure mechanism remains unclear. A prevailing view is that the failure of amorphous carbon film results from the plastic deformation of substrates or strong adhesion between two contacted surfaces. In this paper, using first-principles and molecular dynamics methodology, combining with compressive stress-strain relation, we firstly demonstrate that the plastic deformation induces graphitization resulting in strong adhesion between two contacted surfaces under vacuum, which directly corresponds to the cause of the failure of the films. In addition, sliding contact experiments are conducted to study tribological properties of iron and fluorinated amorphous carbon surfaces under vacuum. The results show that the failure of the film is directly attributed to strong adhesion resulting from high degree of graphitization of the film, which are consistent with the calculated results.

Amorphous carbon films, which are considered to be one type of the next-generation lubricant materials, have gained increasing attention due to their superior tribological properties under vacuum^1^,^2^,^3^. Especially, fluorinated amorphous carbon film against steel ball displays superlow friction (<0.01) under high vacuum^4^. Although these films exhibit excellent tribological properties under vacuum as reported by the most of experimental and computational results^5^,^6^,^7^,^8^, these films would ultimately lapse^9^. To date, most of previous researches merely focus on superlow friction^8^,^10^, however, the intrinsic failure mechanism has been still not well understood.

The prevailing views are that the plastic deformation of the substrates and strong adhesion between two contacted surfaces are considered as two main factors for the failure of the amorphous carbon film. Given that the intrinsic tribological behavior of a film, substrate deformation (elastic, plastic or elasto-plastic) plays a crucial role in governing the overall frictional response of a frictional system. Jungk and Zeng reported that plastic deformation was induced at the large contact stress regimes, the accumulated plastic strain at the film-substrate interface would result in film breakdown, leading to the film removing from the substrate^11^,^12^. On the other hand, the strong adhesion caused by interatomic forces between two contacted surfaces is the most common reason for the high friction and heavy surface damage^13^. Indeed, as reported by most of previous researches^14^,^15^, the initial counterpart would be often covered carbon-riched transfer layers in vacuum. As a result, the high friction and severe wear resulted in the failure of the film, as reported by our previous work^16^,^17^.

Especially, the bonding patterns in the carbon films could affect the tribological performances^18^. Gueorguiev and coworkers theoretically predicted and guided the synthesis of fluorinated carbon films with varying fluorine content based on first principles calculations^19^,^20^. And they pointed out the boding patterns in the fluorinated carbon films, which was conducive to probing the failure mechanism of fluorinated carbon films.

Due to the difficulties in direct observation of the failure processes by in-situ experiments with atomic resolution, it is not trivial to understand the contribution of each mechanism. However, first-principles and molecular dynamics (MD) simulations provide a powerful tool to capture atomic details and gain a deeper insight into the intrinsic failure mechanism of fluorinated amorphous carbon film at the nanoscale. In the present study, according to our experimental results, we choose to study Fe/fluorine terminated diamond (FTD) surfaces in order to illustrate the failure mechanism of fluorinated amorphous carbon films against steel ball under vacuum. This was because that the fluorinated amorphous carbon film surface was often represented by a F-terminated diamond surface following the common practice used in the literature of employing diamond to use as a model to study the amorphous carbon surfaces^21^,^22^. We elucidate the intrinsic failure mechanism of fluorinated amorphous carbon film under vacuum using first-principles and MD simulations. Meanwhile, stress-strain relation had a significant effect on the tribological properties of steel and MoS_2_^23^,^24^, but had not been reported for amorphous carbon film. Here, we characterize the compressive stress and strain along the 〈111〉 direction in order to probe the intrinsic failure mechanism of fluorinated amorphous carbon film. The results show that graphitization under elastic deformation is conducive to obtaining low friction. Conversely, plastic deformation induces graphitization leading to strong adhesion, which directly corresponds to failure of the film under vacuum.

## Results

**Figure 1:**
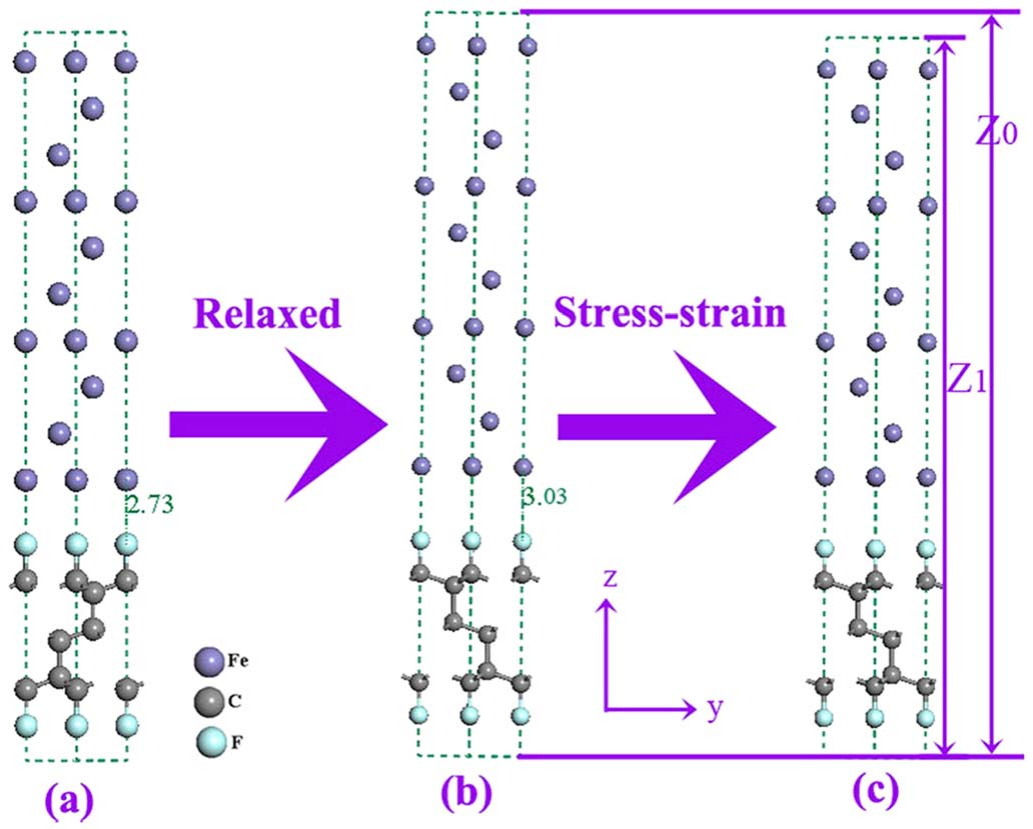
The schematic diagram to visually present interfacial system and direction of application of the strain: (a) the initial configuration (the interfacial distance is 2.73 Å), (b) the relaxed configuration, the stress along *x*, *y* and *z* directions is zero, this configuration could be treated as strain ∈ = 0 (equilibrium position, the interfacial distance is 3.03 Å), (c) the stress and strain is calculated in this configuration by changing the length of Z_0_. The fixed compressive strain could be calculated by the formula: ∈ = (Z_0_ − Z_1_)/Z_0_.

Inversion symmetry is enforced in all cases to ensure that two interfaces are identical and the interfacial energy is uniquely defined. Figure 1 shows the schematic diagram of the calculated progress of strain. An interfacial distance of 2.73 Å is observed in the initial configuration, while the equilibrium configuration (∈ = 0) with an interfacial distance of 3.03 Å can be obtained after the relaxation of the initial configuration (Figures 1a and 1b). By continuously changing the length of Z_0_, meanwhile, fixing the length of Z_0_, the compressive strain can be further calculated through the formula: ∈ = (Z_0_ − Z_1_)/Z_0_ (Figures 1b and 1c).

**Figure 2:**
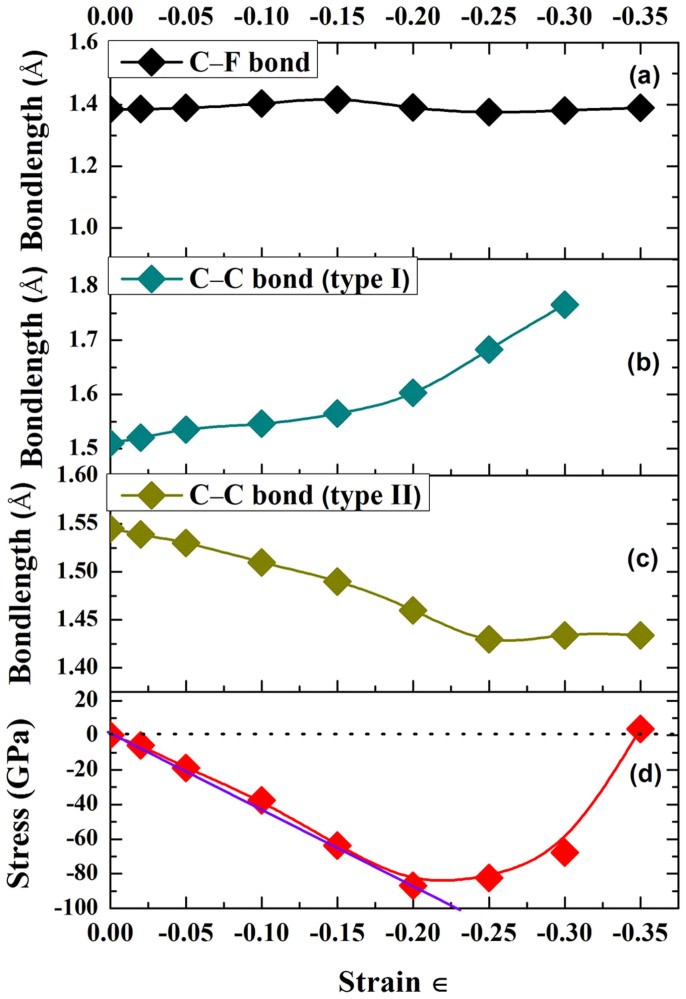
(a) Calculated C-F bond length, (b) and (c) C-C bond length (type I and II), (d) stress-strain curve versus strain of Fe/fluorine terminated diamond system along the 〈111〉 direction. Type I is perpendicular to the direction of the strain, and type II is parallel to the direction of strain.

We have calculated the stress-strain relation of Fe/FTD system along the 〈111〉 direction. Figure 2 shows the calculated C-F, C-C bond lengths and stress versus strain curves of the Fe/FTD system. In Figure 2a, the C-F bond length slightly increases, then decreases, finally remains constant with increasing strain. As shown in Figure 2b, the C-C bond length of type I under compression rises with increasing strain until the diamond structure is broken at the strain ∈ = −0.3 (see Supporting Movie). The C-C bond length of type II under compression decreases when the strain is less than −0.25, then slightly increases with further increasing the strain, as shown in Figure 2c. The ratio of compressive stress to compressive strain can be illustrated as violet line shown in Figure 2d. For the compressive deformation along the 〈111〉 direction, almost linear proportionality up to about 20% compressive strain can be found. Interestingly, a positive stress about 3.72 GPa is obtained at the strain ∈ = −0.35, which is attributed to the lattice distortions of the system (see Supporting Movie).

**Figure 3:**
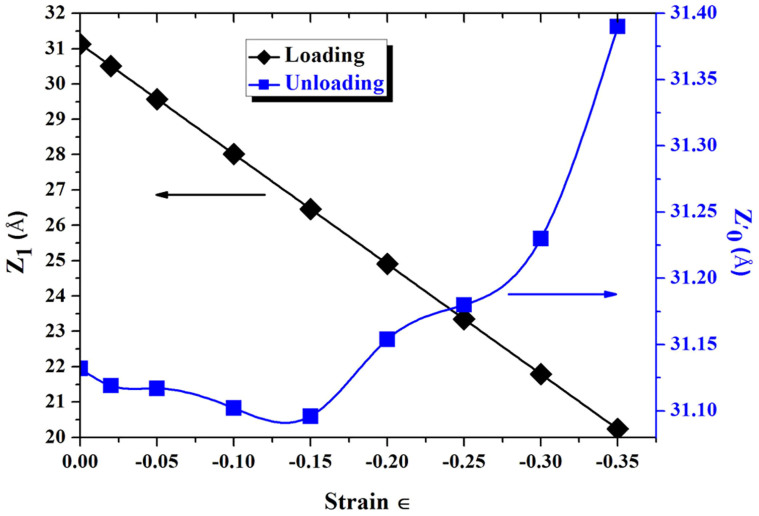
The loading and unloading process curves. The restoration rate (RR) could be calculated from the formula: RR = Z'_0_/Z_1(∈ = 0)_.

It is well-known that if a deformation is elastic, the deformation would be reversible under unloading process. It can be seen from Figure 3 that the restoration rate of the system is more than 99.0% in the range from −0.02 to −0.15 (The restoration rate (RR) could be calculated from the formula: RR = Z′_0_/Z_1(∈ = 0)_). Thus, the deformations are elastic in the range from −0.02 to −0.15. However, the systems are still reversible after strain more than −0.15, but Z′_0_ > Z_1_ (∈ = 0). Even the systems are reversible at the strain ∈ > −0.15, it is unable to determine which deformation sets in. This is because that the primary aim of relaxation processes based on first- principles calculations is to obtain the stable configuration with a minimum energy. The unloading processes of the systems can be seen in Supported Movies.

**Figure 4:**
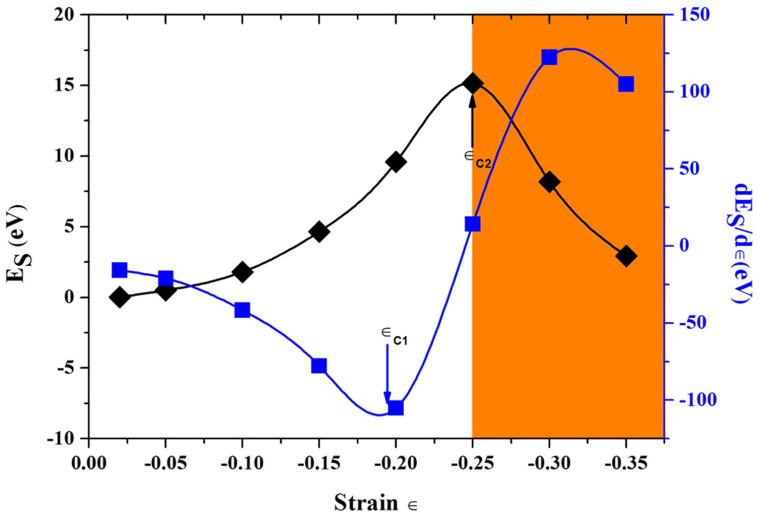
Variation of strain energy and its first derivative with respect to the uniform strain ∈ (E_S_ = E_∈ = *x*_ − E_∈ = 0_, *x* = −0.02, −0.05, −0.1, −0.15, −0.2, −0.25, −0.3, −0.35). Orange shaded region indicates the plastic range. Two critical strains in the elastic range are labeled as ∈_C1_ and ∈_C2_.

As shown in Figure 4, the strain energy first increases at the strain ∈ < −0.25, then sharply decreases when the strain is more than −0.25. In addition, the differences in the derivative of strain energy with different strains are also presented in Figure 4. The values of strain energies are listed in Table 1. Two critical strain values are deduced. The first one, ∈_C1_, is the point where the derivative curve attains its minimum value, indicating that the structure can be compressed under smaller compression for higher values of strain. The second one, ∈_C2_ (−0.25), represents the yielding point. Beyond this point, the plastic deformation sets in. Thus, combining with Figure 3, the deformations are still elastic when the strain in the range −0.15 <∈< −0.25. The deformation is plastic when the strain ∈ is larger than −0.25^25^,^26^. According to the above discussion, the change of strain energy and the increase of stress in Figure 2d after compressive strain (∈ = −0.2) can be attributed to the change of the C-C bond lengths resulting in the modification of the structure, as confirmed by the most of previous researches^27^,^28^,^29^. The fixed slope of the stress-strain curve indicates that the present deformation is an elastic deformation. The calculated compressive elastic modulus in this region is 434 GPa, as shown in Figure 2d.

**Figure 5:**
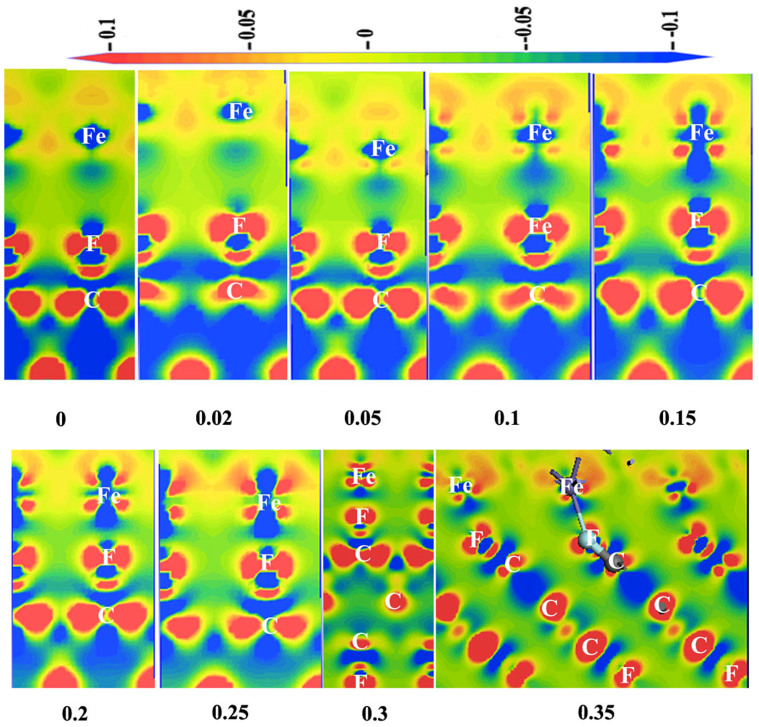
The valence charge density (in electrons/cell) for Fe/FTD system along the 〈111〉 direction at the equilibrium (∈ = 0) and elastic or plastic strain (∈ = −0.02, −0.05, −0.1, −0.15, −0.20, −0.25, −0.3, −0.35).

Next, the atomistic deformation mechanism of Fe/FTD system in compressive deformation is presented. The interfacial valence charge density under different strain should be first examined at the structure of Fe/FTD interface. As shown in Figure 5, the shape of valence charge density of C-F bonds near the interface is varied from the equilibrium value with increasing the compressive strain. Moreover, the valence charge density of F atoms near Fe atoms becomes zero at the strain ∈> −0.3. It indicates that the electronic structure of the system undergoes significant change under plastic deformation. It is interesting to note that the valence charge density of Fe atoms significantly increases from the equilibrium value and shifts to the F atom side.

**Figure 6:**
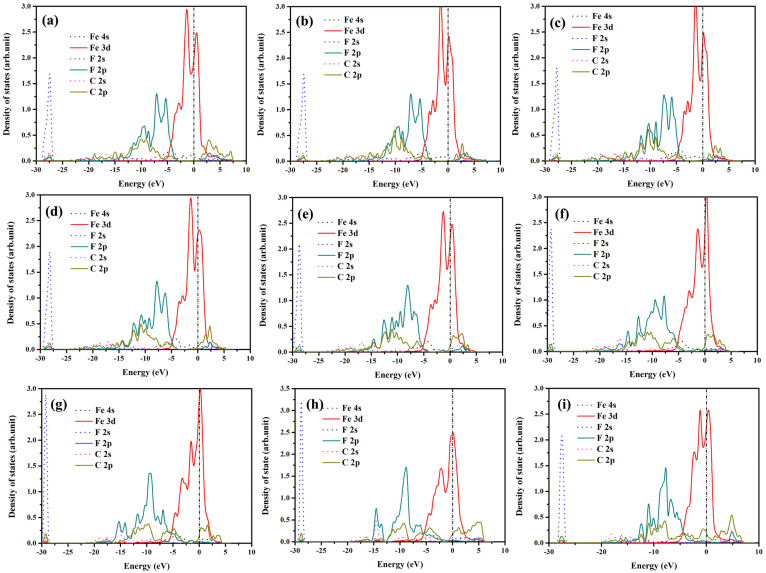
The density of states of Fe/FTD system under different strain∈, (a) 0, (b) −0.02, (c) −0.05, (d) −0.1, (e) −0.15, (f) −0.2, (g) −0.25, (h) −0.3, (i) −0.35. Fermi energy level E_F_ = 0 eV.

To further analyze the constitution of valence band (VB) and conduction band (CB), and understand the change of electronic structures brought about by compressive strain, we have performed density of states for interfacial Fe, F and C atoms, as shown in Figure 6. Figures 6a–6i show that VB consists mainly of F 2s, F 2p, C 2p and Fe 3d states. The hybridization of C 2p, F 2p and Fe 3d states is observed in VB. In Figures 6a–6e, under the elastic deformation, the CB mainly consists of Fe 3d, C 2p, F 2p states. Interestingly, in Figures 6f–6g, F 2p state disappears in CB and C 2p state in VB shifts upward to near the Femi energy level attributing to the effect of compressive strain. And the electrons of F 2p state in CB transform to VB resulting from the compressive strain. However, as shown in Figures 6h–6i, the F 2p state appears in CB again under plastic deformation. Moreover, the electronic density of Fe 3d state decreases. The F 2s state shifts to direction of the lower VB under elastic deformation. however, it is drew to the upper VB under plastic deformation.

According to the above discussion, it can be concluded that the changes of valence charge density and density of state are attributed to structure modifications of the system under compressive strain process. Based on first principles calculations, we obtain several quantitative results. The system possesses good resistance to compressive strain when the compressive strain is lower than −0.25. While the compressive strain increases more than −0.25, the system gradually tends to lose this capacity.

**Figure 7:**
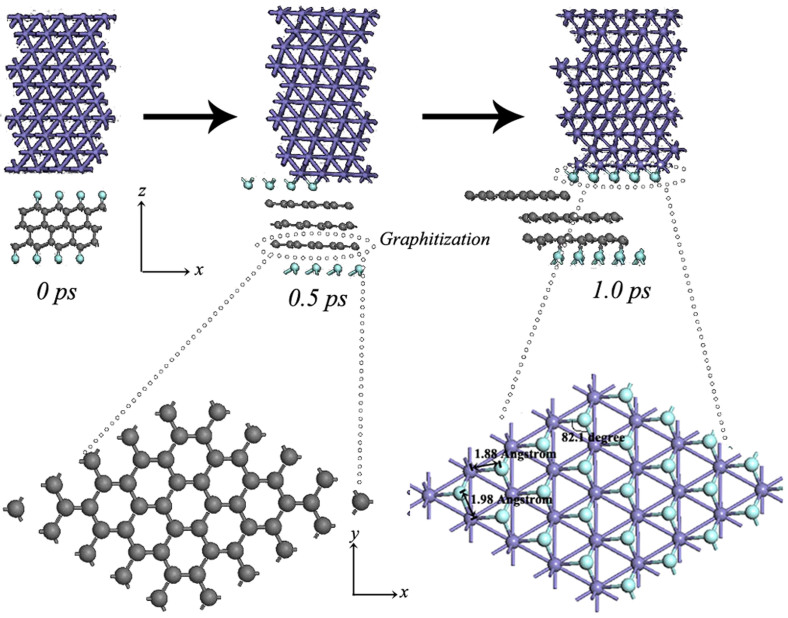
The reconstructed microstructure of Fe/FTD interface system under elastic strain ∈ = −0.15.

**Figure 8:**
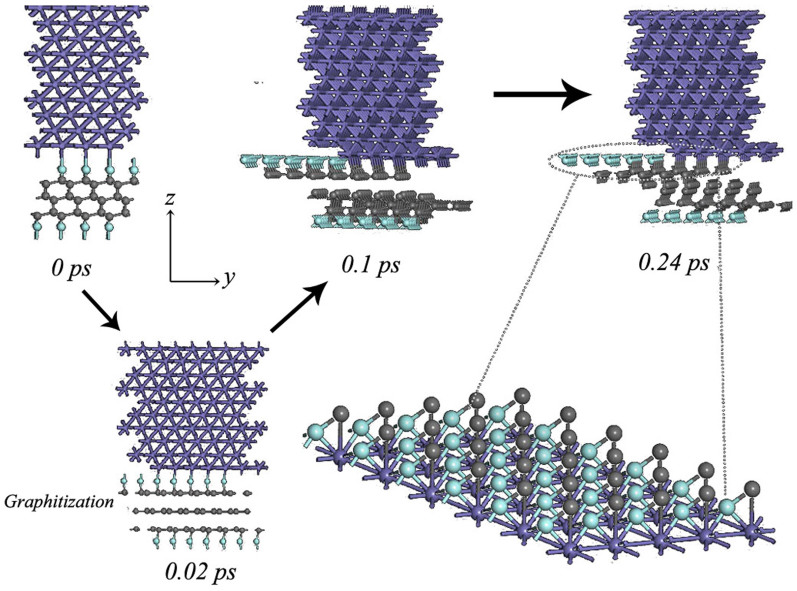
The reconstructed microstructure of Fe/FTD interface system under plastic strain ∈ = −0.3.

The modification of the interface structure can not be well observed during static calculations. Thus, MD simulations are selected to further probe the stress-strain processes under elastic and plastic deformation presented in Figures 7 and 8, respectively. Figure 7 shows the reconstructed microstructure of Fe/FTD interface system under elastic strain ∈ = −0.15. It can be observed in the detailed modification processes that the F atoms transfer to Fe surface and fluorine-free diamond surface slowly transforms to graphite-like structure. In the reconstructed structure (inset in Figure 7), the Fe–F bond distance is 1.98 and 1.88 Å, and the angle F–Fe–F and Fe–F–Fe is 82.1°. Compared with the FeF_2_ crystal, the calculated Fe–F bond distance and F–Fe–F angle values prove to be in good agreement with the experimental value^30^ of 2.05 ± 0.06 Å and 90°. Differently, the calculated F–Fe–F angle and Fe–F bond distance is lower than that of the FeF_2_ compound, because no octahedra is presented in the reconstructed Fe surface. The result indicates that FeF_2_ compound could form under elastic deformation processes. Interestingly, graphitization is detected under elastic deformation processes. Graphitization of the amorphous carbon surfaces with shear within *sp*^*2*^ graphitic basal planes could result in low shear resistance, which is helpful for obtaining low friction in ambient air, water or oil, as reported by Holmberg^31^. However, in our previous study^17^, graphitization has not been conducive to maintain low friction under vacuum. In that work, we have only considered the tribological properties of hydrogenated amorphous carbon films, and have not focused on the condition that how to affect the tribological properties under the coexistence of tribochemical reaction and graphitization. Sen reported that low friction was obtained under the coexistence of tribochemical reaction and graphitization^32^. Thus, we could therefore conclude that the low friction could be obtained under elastic deformation region, due to the effect of FeF_2_ on reducing friction and wear, as reported by the previous study^33^. However, the careful attention is paid to the comparison of the Figure 7. As shown in Figure 8, an interesting result can be observed that in the early stage of the MD time from 0 to 0.02 ps under plastic deformation regime, graphitization is detected, owing to the existence of strong C-F covalent bonds, fluorinated graphite sheet transfers to Fe surface. After 0.02 ps, C atoms shift to Fe side and bond with Fe atoms, and then quickly bond with the rest C atoms in FTD exhibiting strong adhesion between Fe and FTD in a short simulation time. In addition, FeF_2_ compound does not be detected in this stage. Graphitization and strong adhesion between Fe and FTD surfaces is merely observed under plastic deformation regimes. The detailed simulation processes are shown in Supported Movies. Two quantitative results are obtained based on MD simulations. The system could well advoid the adhesion between Fe and fluorinated diamond surfaces under the strain ∈ = −0.15. However, the strong adhesion occurs between two contact surfaces at the strain ∈ = −0.3.

**Figure 9:**
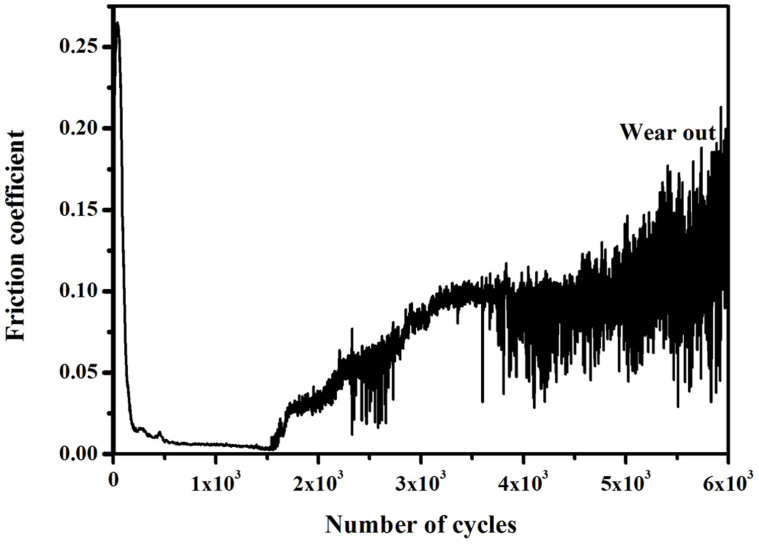
The typical friction coefficient curve of fluorinated amorphous carbon film under vacuum.

Figure 9 shows the friction coefficient (FC) curve of the film sliding against steel ball under vacuum. Only a short superlow FC stage about 1600 sliding cycles is observed. Then, the FC sharply increases until the wear out of the film. According to the analysis of MD simulation under plastic deformation regions, strong adhesion between two contact surfaces could occur in a short time. Thus, the superlow friction could be obtained in the short time. Actually, in our previous study^16^, the short superlow FC stages can be successfully achieved when the amorphous carbon films against SiC and Si_3_N_4_ balls under high vacuum.

**Figure 10:**
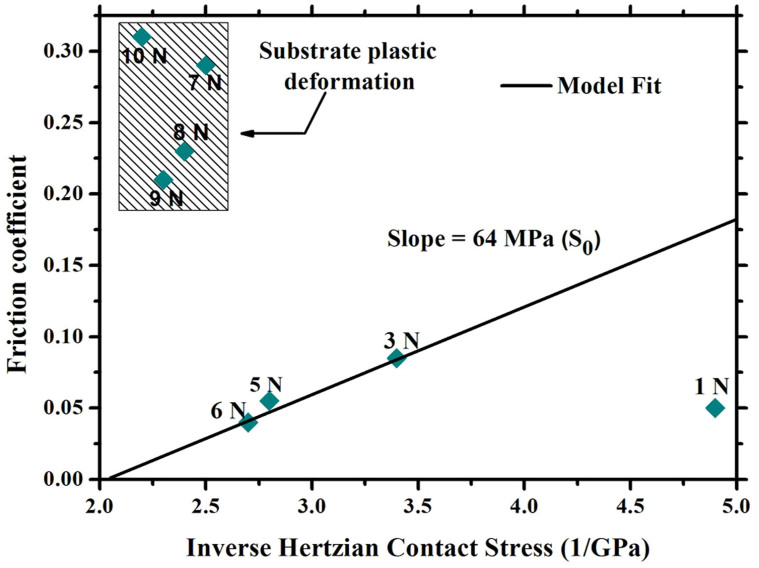
Compilation of all friction data showing two distinct regimes: (i) linear relationship between friction coefficient and inverse contact stress corresponding to interfacial shear (straight line), and (ii) substrate plastic deformation with substantial increase in friction coefficient (rectangle).

**Figure 11:**
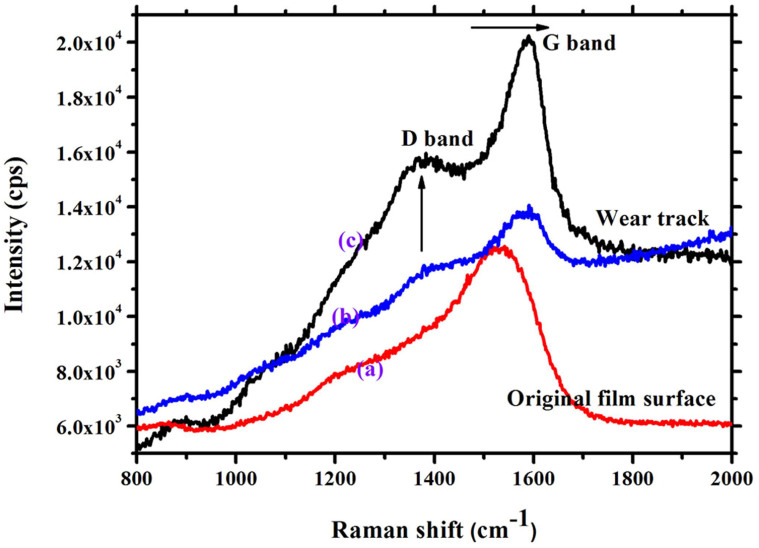
Raman spectra of original film surface (curve “(a)”) and wear track (curve “(b)” and “(c)”) sliding under vacuum.

In order to rule out of the plastic deformation of substrate that leads to failure of the film, all the FCs of the film against steel ball are presented in Figure 10 as a function of the inverse contact stress. The data fall in two distinct regimes. There is a straight line trend where the FC increased linearly with increasing in inverse contact stress. This regime corresponds to no gross plastic deformation in the metallic substrate. The slope (S_0_) of the straight line, which is a measure of the interfacial shear strength of the film, is 62 MPa. It can be calculated by the formula: 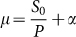
11. *μ* is friction coefficient, *P* is Hertzian contact stress, *α* is a constant^34^,^35^. The second regime of FC values in the rectangle region of Figure 10 corresponds to the plastic deformation of the underlying substrate. Thus, it can be concluded that plastic deformation of the substrate does not occur when the load is set as 2 N during sliding. Subsequently, the microstructure of the film surface and wear tracks during different sliding cycles is detected by Raman spectra presented in Figure 11. With increasing the sliding cycles, the D peak becomes more pronounced and the position of G peak shifts to higher Raman frequency, indicating significant graphitization of amorphous carbon film^36^,^37^. According to above discussion, the failure of the film depends on the high degree of graphitization of the film resulting in strong adhesion between two contacted surfaces. The calculated results are consistent with the experimental observations.

## Discussion

In summary, graphitization was a controversial issue in the tribological field of diamond-like carbon films over the past decades. Most researchers believed that the graphitization was helpful for reducing friction under ambient air and water vapor^38^,^39^. On the other hand, it was reported that high friction and wear resulted from high degree of graphitization under vacuum^17^. Due to the difficulties in direct observation of the graphitization process by in-situ experiments with atomic resolution, however, first-principles and MD simulations provided a powerful tool to capture atomic details and gained a deeper insight into the graphitization process. Here, we systematically investigate the relation between intrinsic failure mechanism and graphitization using first-principles calculations and molecular dynamics simulations. Combining with compressive stress-strain relation, the simulation results reveal that plastic deformation leads to graphitization resulting in strong adhesion, which directly corresponds to the failure of the film under vacuum. Under elastic deformation regions, the low friction would be obtained when the tribochemical reaction and graphitization concurs. In addtion, the responses of bond lengths during the strain processes significantly influence the mechanical properties of the system, as reported by the most previous studies^27^,^28^,^29^. Indeed, in Figure 2, the C-C bonds are sensitive to the compressive strain. The bond-breaking events of C-C bond of type I occur at the compressive strain more than −0.3. The bond softening events of C-C bonds of type II, take place after the compressive strain ∈ = −0.25. In the whole strain processes, the C-F bond lengths remain around 1.4 Å. It indicates that the C-C bonds play an important role in resisting to the compressive stain under elastic deformation regions, rather under plastic deformation regions. The calculated results are in agreement with the experimental observations. Understanding the failure mechanism of fluorinated amorphous carbon film in vacuum is desirable for potential space applications.

## Methods

### Simulation procedure

**Figure 12:**
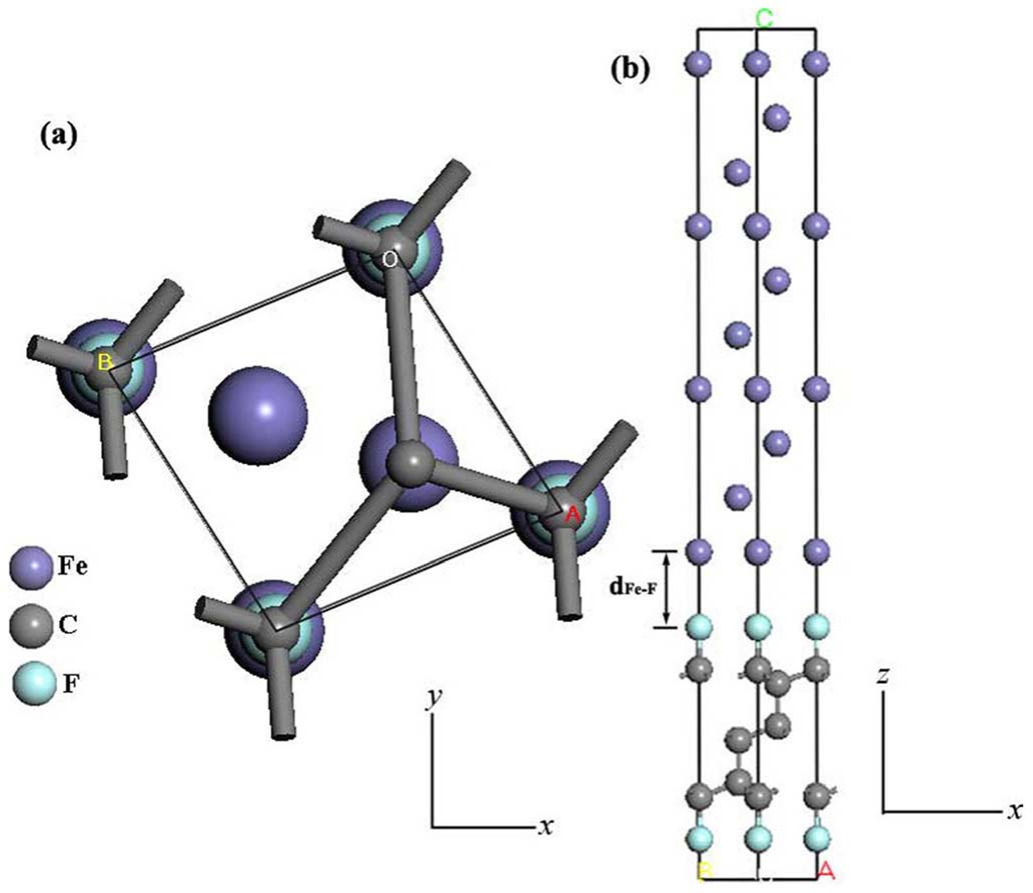
Fe and FTD interface model used in DFT calculations. (a) Top view of the interface registry, where the edge length of the cell is 2.50 Å. (b) Side view of the interface model. *d*_Fe–F_ is the distance between the Fe and F atom at the interface.

The CASTEP module in the Materials Studio 5.5 program of Accelrys Inc was used to calculate the ground state energy and geometry of each interface. Zilibotti and coworkers^40^ investigated the nanotribological properties of passivated diamond surfaces using the generalized gradient approximation (GGA) in the form of the Perdew–Burke–Ernzerhof (PBE)^41^,^42^,^43^. They found that the values obtained from the PBE approximation were in closer agreement with experiments. Thus, we adopted this approximation in the following calculations of Fe and diamond interfaces. Our calculations were fully converged with respect to the size of the basis set. A plane-wave cutoff of 360 eV and Monkhorst-Pack *k*-point meshes with a density of (7 × 7 × 1) were employed throughout. The electron-ion interactions were described by ultrasoft pseudopotentials^44^. A Fermi smearing of 0.1 eV was utilized^45^. The convergence criteria for structure optimization and energy calculation was set to MEDIUM quality with the tolerance for SCF, energy, maximum force, maximum displacement and maximum stress of 2.0 × 10^−6^ eV/atom, 2.0 × 10^−6^ eV/atom, 0.05 eV/Å, 2.0 × 10^−3^ Å and 0.02 GPa, respectively. In this article, the tribological surface of amorphous carbon film was represented by a diamond surface such as (111)^46^. The Fe (111)-1 × 1/fluorine terminated diamond (FTD) (111)-1 × 1 interface was used in all calculations. Fcc Fe was selected to minimize the lattice mismatch of Fe (111)-1 × 1/FTD (111)-1 × 1 interface, this corresponded to an average lattice mismatch of 1%. After geometrical optimizations, the lattice parameters of fcc Fe are 3.40 Å. They were in well consistency with experimental value of 3.45 Å^47^. It indicated that the calculation methods were reasonable and the calculation results should be authentic. A slab model is used to simulate the geometries of the Fe and FTD surfaces, as shown in Figure 12. A slab with ten and six layers would be adequate for the surface stability of Fe and FTD surface^32^. The calculation methods of compressive strength were reported in most previous studies^29^,^48^. At each step, the fixed and unfixed compressive strain was applied in the 〈111〉 direction, and then the structural parameters were relaxed until the stress tensors orthogonal to the applied stress less than 0.02 GPa. For the 〈111〉 direction, there were two different types of C−C bonds under deformation. One type was perpendicular to the direction of the stress (denoted as type I), and the other type was parallel to the direction of stress (denoted as type II). In this way, a series of compressive stresses corresponding to the incrementally applied compressive strains could be obtained. In MD simulations, the super-cell (4 × 4 × 1) contained 144 Fe, 96 C and 16 F atoms in the interface model. MD simulations were conducted on each interface model with the constant volume and energy (*NVE*) method at an initial temperature of 300 K. The *NVE* method was chosen to allow both the temperature and stress of the system to change under the external force. A MD time step of 1.0 fs was used for the simulation model. The total MD simulation time was set as 1.0 ps.

### Experimental procedure

Raman spectra of the as-deposited coating were obtained using a Horiba Jobin Yvon LABRAM-HR800 spectrometer with an excitation wavelength of 532 nm. The typical spectrum was recorded in the range of 800–2000 cm^−1^ and data acquisition time was 30 s. The frictional behavior of the sample was performed on a vacuum tribo-meter with a frictional force sensor with accuracy rating of 0.01%, using a rotational ball-on-disc mode. Tribological tests were done under vacuum ranged from 1 × 10^−3^ to 5.0 × 10^−4^ Pa. The rotational speed was 300 rev min^−1^, and the rotational radius was set to 4.0 mm. The corresponding linear speed was about 0.125 m s^−1^. The counter face was standard GCr15 steel ball with 4 mm diameter. The normal load was set as 2.0 N, corresponding to a theoretical initial Hertzian contact pressure of 0.62 GPa.

## Author Contributions

L.W., R.Z. and Z.L. conceived the first-principles and molecular dynamics simulations and R.Z. carried them out. All authors discussed the results and wrote the paper.

## Supplementary Material

Supplementary Information Video S1

Supplementary Information Video S2

Supplementary Information Video S3

Supplementary Information Video S4

Supplementary Information Video S5

Supplementary Information Video S6

Supplementary Information Video S7

Supplementary Information Video S8

Supplementary Information Video S9

Supplementary Information Video S10

Supplementary Information Video S11

Supplementary Information Video S12

Supplementary Information Video S13

Supplementary Information Video S14

Supplementary Information Supplementary Information

## Figures and Tables

**Table 1:** The values of strain energy (E_∈_ and E_S_) under different compressive strain, where E_∈_ is the strain energy calculated at the strain ∈. E_S_ is the strain energy calculated by substracting the total energy of the strained system from the equilibrium total energy

Strain	Strain energy (E_∈_) (eV)	Strain energy (E_S_) (eV)
0	−10920.07	0
−0.02	−10920.04	0.03
−0.05	−10919.57	0.5
−0.1	−10918.27	1.8
−0.15	−10915.42	4.65
−0.2	−10910.48	9.59
−0.25	−10904.91	15.16
−0.3	−10911.90	8.17
−0.35	−10917.15	2.92
