# Supplementary material for: Probing the intrinsic failure mechanism of fluorinated amorphous carbon film based on the first-principles calculations
Source: Sci Rep. 2015 Mar 24;5:9419. doi: 10.1038/srep09419 (PMC4371838; doi:10.1038/srep09419)
Supplement: Supplementary Information [file srep09419-s15.doc]

**Supporting Information**

**Probing the intrinsic failure mechanism of fluorinated amorphous carbon film based on the first-principles calculations**

Ren-hui Zhang^1,2^, Li-ping Wang*^1^, Zhi-bin Lu^1^

^1^State Key Laboratory of Solid Lubrication, Lanzhou Institute of Chemical Physics, Chinese Academy of Science, Lanzhou 730000, China, ^2^University of Chinese Academy of Sciences, Beijing 100049, China.

*Correspondence to L.P.W. ( lpwang@licp.cas.cn)

Tel: +86 931 4968080

Video legends for the movies:

- Video S1 ......................................................................................................... Page S2

- Video S2 ......................................................................................................... Page S2

- Video S3 ......................................................................................................... Page S2

- Video S4 ......................................................................................................... Page S2

- Video S5 ......................................................................................................... Page S2

- Video S6 ......................................................................................................... Page S2

- Video S7 ......................................................................................................... Page S2

- Video S8 ......................................................................................................... Page S2

- Video S9 ......................................................................................................... Page S2

- Video S10 ........................................................................................................Page S2

- Video S11 ........................................................................................................Page S3

- Video S12 ........................................................................................................Page S3

- Video S13 ........................................................................................................Page S3

- Video S14 ........................................................................................................Page S3

Video S1:

The compressive strain process at the strain ∈ = 0 using first-principles calculation.

Video S2:

The compressive strain process at the strain ∈= −0.02 under unloading using first-principles calculation.

Video S3:

The compressive strain process at the strain ∈= −0.05 under unloading using first-principles calculation.

Video S4:

The compressive strain process at the strain ∈= −0.10 under unloading using first-principles calculation.

Video S5:

The compressive strain process at the strain ∈= −0.15 under unloading using first-principles calculation.

Video S6:

The compressive strain process at the strain ∈= −0.20 under unloading using first-principles calculation.

Video S7:

The compressive strain process at the strain ∈= −0.25 under unloading using first-principles calculation.

Video S8:

The compressive strain process at the strain ∈= −0.30 under loading using first-principles calculation.

Video S9:

The compressive strain process at the strain ∈= −0.30 under unloading using first-principles calculation.

Video S10

The compressive strain process at the strain ∈= −0.35 under loading using first-principles calculation.

Video S11:

The compressive strain process at the strain ∈= −0.35 under unloading using first-principles calculation.

Video S12:

The tensile strain process at the strain ∈= +0.05 using molecular dynamics simulation.

Video S13:

The compressive strain process at the strain ∈= −0.15 using molecular dynamics simulation.

Video S14:

The compressive strain process at the strain ∈= −0.30 using molecular dynamics simulation.
